# Selections and Abstracts

**Published:** 1902-03

**Authors:** 


### SELECTIONS AND ABSTRACTS.

---

### SMALLPOX.

*Diagnosis.*—In a leading article* it is stated that the specific eruption of variola is often preceded and in rare cases accompanied by hæmorrhagic or erythematous eruption which is generally spoken of as a rash. This symptom is very confusing, and has doubtless led to many grave errors. These rashes are very different from the smallpox eruptions, and may easily suggest a diagnosis which may prevent the recognition of the disease when its true characters are known.

The period of invasion in smallpox—marked by fever, chills and vomiting—commonly covers three or four days. The rash may appear early or late in this period. Osler says that it is more frequent in some epidemics, and it occurs in from 10 to 16 per cent. of cases.

H. Roger and Emile Weil have studied the rash in 978 cases, and have determined that the number of cases with initial rash bore some relation to the severity of the disease. Their figures for the total percentage of those having a rash is nearly the same as Osler's—18.4 per cent. The cases of varioloid gave the smallest number, 12.8 per cent.; the discrete and confluent cases, 21 per cent.; the confluent, 20.7 per cent.; the hæmorrhagic, 47.5 per cent. Of the 40 cases of hæmorrhagic smallpox, all died; and, of these, 19 had initial rashes.

In speaking of the form of the rash Osler says two types can be distinguished—a diffuse scarlatinal and a macular form—either of which may be associated with petechiæ. The scarlatinal rash may come out on the second day and be as diffuse and vivid as a true scarlatina. The macular form may be identical with that of measles.

Roger and Weil distinguish six forms of rash—erythematous, urticarial, morbiliform, scarlatiniform, purpuric and astacoïde.

* Medicine Oct., 1901.

The erythema occurs in plaques of a uniform-red color, and disappears upon pressure. The urticarial form occurs in white plaques, reddened at the edges and slightly raised, which generally itch. More frequent than the two preceding is a macular form of erythema: the spots slightly elevated, with a velvety feel, the reddened areas separated by healthy skin. This form exactly resembles measles. The scarlatiniform rash exactly resembles that of scarlet fever. The skin in the affected region is a dark red, which disappears only in part under pressure. The purpuric rash is identical with that of ordinary purpura.

The astacoïde, or lobster-like, rash is identical with the hæmorrhagic form of smallpox. It is found in cases which are apparently benign, and in those in which the early symptoms are severe. Its appearance is a certain indication of a fatal termination. Unlike the other rashes, it is general, excepting the face. It is not a generalized purpura, but has a uniform tint—a diffuse hæmorrhagic suffusion.

These eruptions may be associated in the same patient. In the 171 cases of Roger and Weil 146 had a single eruption. In 25 other cases the eruptions were associated, in all giving 51, or 197 rashes in 171 cases.

Herman Spalding* remarks that an absolute diagnosis of smallpox cannot be made before the eruptive stage, except in the hæmorrhagic form. In all these mild cases there is a general conformity to the history and course of the typical case of smallpox. There is the prodromal symptom, with the subsidence of fever when the eruption appeared. But chickenpox is sometimes preceded by prodromal symptoms, and chickenpox attacks adults, and the prodromal symptoms in smallpox are sometimes irregular and slight; so that one is compelled in some cases to make the diagnosis on the character of the eruption alone, as presented to the eye. The diagnosis can be made, for there is a difference in the appearance to the eye and the clinical course of the development of the skin-lesion. The smallpox lesion has a deeper origin than chickenpox. The papule rests on the cutis vera, and by inflammatory extension

* Jour. Amer. Med. Assoc., Aug. 3, 1901.

this tissue is implicated. Chickenpox lesions have only the outer layer of the skin for a covering. This covering is fragile, easily broken, and is broken from the first to the third day of its growth. The vesicles or pustules of chickenpox, no matter how robust, will not resist accidental violence or natural decay more than four days, except on the hands and wrists for an adult negro, where the covering of the pustule is so thick as to resemble or merge into the life-history of smallpox lesions. The external layer of skin on an adult negro is thick, and the eruption of varicella here looks and feels like the papule of variola, but an examination of the lesion on other parts of the body will aid in determining the nature of the disease. Measles is a papular disease, and the first day of the eruption is often mistaken for variola. In measles the fever is highest on the day of the eruption, while in smallpox this is a time when the temperature drops to the normal usually.

In making a diagnosis of variola there are some prodromal symptoms in the mildest cases, if the physician is successful in getting the truth out of the patient; when the eruption appears the patient will always feel better, unless there is present some intercurrent disease, and the fever will subside as the eruption appears. One should look for the early appearance of the eruption on the vascular parts: on the face, in the throat, on the wrists and hands, on the foreskin and glans penis. If a papule is found on the ears, it will help in the diagnosis. Too much dependence should not be placed on finding umbilications of the vesicles in the mild cases, as it is often absent.

F. Montizambert* states that the extreme mildness of the present disease has rendered it unusually difficult to handle, control, and stamp out.

Severe cases of smallpox are, as a rule, too ill to leave their bed, and are eager to obtain medical attendance. This leads to notification, isolation, disinfection, and the vaccination of those who have been exposed to the infection.

But this type offers more difficulty to the Public Health authorities. There is, as a rule, but little initial fever, a very sparse discrete eruption, and no secondary fever. The patient is not usu-

* Brit. Med. Jour., May 11, 1901.

ally confined to bed, or even to the house, and no medical man is called in. In the country parts it is very generally regarded and spoken of as a chickenpox or German measles. In many of the lumber camps it goes by the name "cedar itch." Those affected by it go to their work or their business, travel in public conveyances, go from one part of the country to another, not only in the period of incubation, but often also in the early period of the eruption, and thus spread the disease generously and widely.

That this disease is smallpox is questioned by many. The following facts, however, in connection with it give a reasonable assurance that it is smallpox, and not chickenpox. It attacks adults quite as often as, or indeed more often, than children. It attacks the unvaccinated or those who have not been vaccinated for some time. It does not attack those who have been recently successfully vaccinated. Every here and there a susceptible person develops a severe confluent or even fatal case.

*Etiology.*—An editorial* states that the recent observations of Roger constitute a valuable addition to our knowledge of the placental transmission of disease to the fetus. A series of cases in 11 pregnant women was observed in his small-pox service at Aubervilliers, all of whom gave birth to children who appeared to be absolutely well, but who all presented marked subnormal temperature: in one instance 28° C., in another 30° C., and in another 31° C. Seven of the children shortly developed marked manifestations of smallpox, the symptoms appearing in a typical manner, beginning with an elevation of temperature, followed by the characteristic cutaneous eruption; 3 of the remaining 4 showed intense infection, the only symptoms being jaundice and subnormal temperature, with death four, six, and eleven days subsequent to birth. One child developed a scarlatiniform eruption, and only 1 of the eleven survived, the others dying in from two to three days after the development of the eruption. Next to syphilis the exanthemata are most prone to affect the fetus *in utero*. The association of the disease with the pregnant condition reacts unfavorably upon the mother. Maternal death is not infrequent under these circumstances, and in every instance in which a fatal termination

*Phila. Med. Jour., Sept. 7, 1901.

does not supervene the mother passes through a most critical illness.

Donald B. Pritchard* had an epidemic in charge in which there were 1758 cases of smallpox. There was one effect that was very marked, namely : A great many women in the first few months of pregnancy aborted. One fetus was in about the third month of its development, and had quite a number of well-marked vesicles. One woman in confinement at full term had been a victim of smallpox for about ten days, and the child was literally covered with small vesicles. The skin was involved to such an extent that it was hardly thought that it could live. The vesicles all disappeared without becoming pustular, and the child made a smooth recovery.

*Prophylaxis.*—In a recent epidemic of 450 cases J. W. Preston† in touching the efficacy of vaccination, can recall but 1 instance of the possessor of a well-pitted scar developing the disease, although a great number were exposed. At Maybeury, a man, wife, and three boarders were equally exposed ; all except the husband and one boarder had been successfully vaccinated. These two latter promptly developed clearly-marked cases of the disease, while the remaining three were quarantined in the same house, and were almost constantly together in the same room for a period of three weeks, and failed to develop it. Could every individual be vaccinated, and this repeated at proper intervals, smallpox could be placed in the same category as the mild exanthemata.

Donald B. Prichard‡ has proved that vaccination protects against smallpox. Of 1758 cases of which he has record, but 17 showed evidence of having at any time been successfully vaccinated. Of the 17 referred to, but 3 had been recently vaccinated, and the disease was so modified in these that the diagnosis was made very difficult. Two of the cases had but one well-marked smallpox vesicle, the third about a dozen. The other 14 had been vaccinated at periods varying from ten to over sixty years previonsly.

There were 152 houses in which there was only 1 case of small-

*St. Paul Med. Jour., Oct. 1901.

†Va. Med. Semi-monthly, Sept. 13, 1901.

‡St. Paul Med. Jour., Oct , 1901.

pox, and in each house that case occurred in the only unvaccinated member of the family. There were hundreds of houses in which every unvaccinated member of the family contracted the disease, and all the vaccinated, irrespective of how old the vaccination was, escaped. It is true that there were a few, a very few, families in which some of the unvaccinated escaped. The disease respected neither the old nor the young, the line being drawn, and that sharply, between the vaccinated and the unvaccinated.

G. A. Kennedy* notes the recent out-break of the disease in the Northwest Territory: An outbreak which was wide-spread. Dr. Patterson, Quarantine Officer for the Dominion Government, is satisfied that there had been 1,500 cases. The greatest number of cases occurred among the French half-breeds, who had never been vaccinated. Indians on reserves did not suffer to any great extent, as annual vaccination is the rule. Not one case was seen or heard of among Galicians, Doukhobors, or Roumanians, which is due to the fact that compulsory vaccination was the rule in youth, and then they had been revaccinated on their recent passage across the Atlantic and at Halifax.

An editor† notes that though mild scarlet fever and mild smallpox resemble each other in spreading the disease by means of unrecognized cases, and in the consequent comparative futility of hospital isolation as a preventive measure, they differ in the one essential particular that against smallpox the individual can protect himself by vaccination, while scarlet fever has no similar shield. If there were no exceptions to the very trivial character of the variola in the present American out-break, the question of risking attack might conceivably be open to discussion; but seeing that confluent and hæmorrhagic smallpox may and does result from infection derived from the mildest cases, the only safety is to be found in vaccination.

Louis Leroy‡ is convinced that glycerinated lymph is by far the best method of inducing the vaccine disease. To obviate the objection that it is viscid, takes a long time to dry, and when exposed

*Dominion Med. Monthly, Sept., 1901.

†Brit. Med., Jour., Sept. 21, 1901.

‡Jour. Amer. Med. Assoc., Aug. 3, 1901.

on the surface is liable to be contaminated by foreign substances, which readily adhere to it, the hypodermic needle may be used, introducing the vaccine underneath the skin. A solid-piston hypodermic syringe is used, and, if a large number of vaccinations are to be performed, the barrel of the needle may be filled with the glycerinated lymph. A short and rather large caliber needle is most satisfactory. The skin is cleansed in the usual way, and a drop of the lymph injected with the concave surface of the needle downward. As a rule, a single drop is sufficient, though a larger quantity is not harmful. The advantages of the intradermic method are claimed to be freedom from contamination, absence of pain (an advantage in young children), and simplicity of application. The number of successful results is greater than where simple scarifying is used.

H. M. Bracken* says vaccine is frequently spoiled by not being kept in proper temperatures, as it is frequently being shipped in cans which are too hot, and subsequently kept in warm offices. Personally, all vaccine is kept in an ice-box, but not frozen, and good results have been obtained.

*Treatment*—If fever is high and pain severe, S. S. Watson† advises tablets of codeine compound with, if needed, Norwood tr. veratrum viridi. Saline laxative should be given as indicated. After the appearance of the eruption a daily bath of 1 to 4000 bicloride is of value until the pustular stage; then 1 to 2000 bichloride. When the pustules or vesicles begin to dry, the surface is to be kept well covered with carbolized vaselin. For angina, the throat should be kept sprayed with 1 to 4000 bichloride solution, or chlorate-of-potash and carbolic acid solution.

Louis Leroy‡ says that, upon the outbreak of one or more cases in a locality, a competent physician should be placed in charge and be given as nearly as possible absolute power. The physician in charge should be in communication with the board of health, preferably by means of telephone from the infected district. The strongest possible police cooperation should be obtained, and vigorous

*Dominion Med. Jour., Sept., 1901.

†Texas Med. News, Sept., 1901.

‡Jour. Amer. Assoc., Aug. 3, 1901.

legal prosecution should be instituted at once upon the least willful infringement of quarantine regulations.

In order to avoid as much as possible all kinds of reports and rumors, it is a satisfactory plan to make a daily official report to the newspapers, stating the exact condition of affairs as nearly as possible, without exaggerating or belittling any feature of the situation.

The question of treating the cases in their own homes must in many cases be considered seriously. In the finer houses this can frequently be done with some degree of satisfaction,

As soon as the authorities are informed of a suspicious case, it should be isolated completely for a few days, until further development of the symptoms render diagnosis certain. During this time no one but the physician in charge and the nurse should, under any circumstances, be allowed to enter the room.

In large cities it is generally an easy matter to obtain a large building for the organization of a pest-house. In the less densely populated locality, however, tents offer the most convenient and efficient means of isolation. If possible, two large tents and two or three smaller ones should be obtained. The two large ones can be utilized, respectively, for male and female patients. One of the smaller ones as a kitchen and commissary department, another for the physician in charge and his assistants, in which may also be kept the medical stores, and, if possible, above all things, a telephone. The tents should be floored with matched boards or sawdust to a depth of one or two inches, should be provided with good flies, and be well ditched. If possible, they should be upon high, sandy ground, and not within three hundred yards of other residences. If the weather be cold, they can be easily heated by small stoves placed near the center of the tent.

As soon as the patient leaves his house, whether recovered or removed to a hospital, the premises and all articles which have in any way come in contact with him should be disinfected. Everything possible should be burned. All wooden articles, the floor, walls and ceilings should be thoroughly sponged with a 1 to 1000 solution of bichloride of mercury.

When a patient is ready to leave the hospital, he is made to thoroughly bathe himself and shampoo his hair. He resumes his clothing, which has been sterilized thoroughly. A large dry-goods box may be rendered air-tight by coating the inside with a mixture of equal parts of resin and paraffin, which is melted and poured into all cracks and crevices.

The clothes may be placed in this and sufficient formalin added to moisten them, when the lid will be closed and they are allowed to remain for twenty-four hours. This has always proved satisfactory and does the clothes no injury.—*Monthly Cyclopedia of Practical Medicine.*

---

### SCARLATINAL NEPHRITIS AND ITS TREATMENT.

The frequent occurrence of nephritis as a complication of scarlet fever and the grave character of the symptoms present in many cases certainly justify a discussion of the phenomena and a consideration of some of the more important features in the treatment.

In some years not more than 5 per cent. are affected, in others, on the contrary, nearly one half of the patients show the characteristic changes in the urine.

During the first week, particularly at the height of the eruption, but with the temperature considerably elevated, the urine is scanty, high-colored, and traces of albumin appear. The latter, the result of the cloudy swelling of the renal epithelium, is transient and not important. During the period of desquamation in the second or third week and sometimes later, the urine contains albumin and casts, a genuine nephritis having developed. The condition differs from that described in the first instance. The onset may be insidious indeed; it may be so gradual and mild that it is frequently overlooked; the urine is examined as a routine plan. No matter how mild the course of the eruption the routine examination of the urine must not be neglected. Not infrequently the mildest cases of scarlet fever are followed by severe and even fatal renal troubles.

In other cases the nausea, anemia, headache, apathy, with or without slight edema of the face and extremities, direct attention to the kidneys. The urine is diminished and contains an excess of urates; at times it is smoky, more or less bloody in appearance or no special change in color is noticed.

In mild cases serious symptoms may arise at any time. Not infrequently during epidemics edema of the face and limbs with marked anemia may be present without any evidence (chemical or otherwise) of nephritis. The trouble then is due to the failure of the circulation attending the weakness of the heart, the result of more or less severe myocarditis.

In bad cases the symptoms come on suddenly or they may have been preceded for a short period by vomiting, headache, etc. Small quantities of dark and bloody urine are excreted or suppression may exist. Vomiting, convulsions and coma may lead to a fatal termination in consequence of the acute uremia.

Ordinarily the condition manifests itself in from ten to twenty days after the period of desquamation; Jacobi has observed its occurrence as late as the thirty-seventh and fifty-second day. Cephalagia, more or less intense, is a common symptom. Disturbances of respiration, great dyspnea, with or without bronchopneumonia, edema of the lungs or evident thoracic complications are of frequent occurrence and must be looked upon as uremic in origin.

Vision may be defective, either functionally or due to anatomical changes in the deeper structures of the eye. The blindness is usually temporary.

With the edema of the body generally, local effusions into the serous sacs (pleural, peritoneal, etc.) may occur. Endo- or pericardial complications may arise. Frequently the original throat and eruptive symptoms have been mild or possibly overlooked, and attention is not directed to the patient until edema, pallor, persistent vomiting or other uremic manifestations lead to an examination of the urine. An inspection of the hands and feet will reveal more or less pronounced desquamation, thereby clearing up the etiology.

In some cases an early manifestation of uremia is noticed in the change in the temper or disposition of the child, iritability may show itself, extreme restlessness occurs or the patient may be frightened, alarmed or greatly depressed. Fever is common; edema of the feet, face and body generally may or may not be present. Now and then, particularly when the patient is kept in bed, edema of the scrotum or back first directs attention to the kidneys.

In the treatment of scarlet fever cases the parents should be given to understand that the physician's duties are not terminated when the rash disappears, the temperature subsides, and throat, glandular or ear troubles have been relieved. During the stage of desquamation the great danger is that of a complicating nephritis.

As a routine plan the urine ought to be examined daily. Rest in bed should be insisted upon, particularly in mild cases. An even temperature (not over 72°) should be maintained whenever possible; flannels, light or medium, should be worn according to the season; a fluid diet kept up and water given at regular intervals.

The failure to observe proper precautions is due in some cases to neglect or ignorance, in other instances mild cases are apt to be regarded as " scarlet rash," so-called, and therefore supposed to be free from the danger of kidney lesions.

If the case be seen at the beginning, with scanty and high-colored urine, plain or carbonated water, with or without small quantities of bitartrate of potash or other saline given at frequent intervals in small amounts will soon increase the flow and abort the trouble.

The morbid substances circulating in the blood may be eliminated through the kidneys, the skin or the intestinal tract. It should not be forgotten that there is a marked antagonism between the skin, the bowels and the kidneys; great activity on the part of the functions of one, lessens the secretions by the others. Too often this is forgotten, and prolonged sweating, brisk purging, etc., are practised at one and the same time; of course the result is disappointment.

In the acute inflammatory condition of the kidney, the result of the toxins of scarlet fever, with or without edema, the organs are engorged with blood and the tension in the vessels is increased; under such circumstances no direct irritant to the renal tissues should be administered. The indication is to relieve the engorged capillary vessels of the glomeruli, to restore the renal secretion without adding to the congestion. This can be accomplished in a physiological way. The flow is best favored by a liberal use of water, with or without cream of tartar or the vegetable salts of potash. The latter are very apt to upset the stomach ; if so they must be discontinued.

It is contended that the fibrinous plugs filling the tubules are dissolved by alkaline urine. For this reason the use of the vegetable salts of potassium is favored by many. As a matter of personal experience, water in the beginning seems to act best; later on salines may be added. If the oliguria be pronounced or uremia threatening a normal salt solution, temperature 105° to 110°, may be introduced high up in the rectum through a colon tube (enteroclysis). The water will be absorbed, the vasomotor constriction is lessened, the toxines are diluted and their expulsion is favored by the increased elimination of fluids through the natural channels.

Many favor the subcutaneous use of water by the method of hypodermoclysis. With this the writer has but little practical experience. He has seen cases in convulsions, in coma, etc., do well by giving water in small and frequent doses per mouth with or without colonic flushings.

Digitalis is frequently employed. Aside from its irritant effects upon the stomach its use is contraindicated, as one of its alkaloids (digitoxin) is a decided irritant to the kidneys.

Furthermore digitalis increases the force of the pulse while it diminishes its frequency ; at the same time the vessels are contracted. In scarlet fever the heart muscles are weakened, the blood tension is increased and the renal vessels are already engorged; digitalis, consequently, ought not to be employed in the acute cases.

Calomel in fractional doses (gr. 1-20 to gr. 1-10) every hour serves a useful purpose. It acts as a sedative to the stomach, is diuretic, has a laxative effect and finally reduces the arterial tension. It may be continued for days and days ; only exceptionally is its use followed by stomatitis. The existence of a prior ulceration or follicular stomatitis is of course a contraindication to its employment.

The aluminum carbonate in small, repeated doses is useful in cases of severe dyspnea or threatened pulmonary edema, provided the stomach is not irritable. Dry-cupping of the chest may be employed at the same time. Strychnine is an excellent heart tonic in these cases and may be given subcutaneously or per mouth. Later on the anemia requires iron, either the tr. fr. chlor. or syr. fer. iodid in proper doses. The unpleasant taste of the latter is best covered by the syr. rub. idaei.

If convulsions occur hot-packs, chloral per rectum and salt water irrigations are to be employed.

Water may be administered in small quantities per mouth in addition to the saline solution per rectum. In coma the subcutaneous use of sodium caffeine benzoate in good doses braces up the heart, arouses the patient from the stupor and sets up diuresis.

Uremic blindness, whether functional in character or due to retinal inflammation, disappears as the nephritis subsides. Renal hemorrhages are best treated by hot applications over the loins, mustard plasters and absolute rest. Gallic acid is advised, but its taste is objectionable. Suprarenal extract or its alkaloid, adrenaline might be tried.

Hydrothorax and empyema require appropriate treatment. It is surprising how quickly the amount of urine increases when the intrathoracic pressure is relieved. The anorexia incidental to the disease and the nausea due to the renal lesion makes the question of nutrition one of difficulty. Only small quantities of liquid food at frequent intervals are to be allowed. Broths, clear soups, equal parts of milk and oatmeal water in ʒi doses may be tried and the quantity increased as the digestion improves.

Though aware of the fact that alcohol is contraindicated in nephritis in bad cases with frequent vomiting and dry tongues, etc., ʒi doses of cold champagne and ice given every ten to thirty minutes have tided the patient over the danger point.

Hot packs or hot-air baths may be advantageously used when the edema is marked, the dyspnea excessive, or pulmonary edema is impending or present.

To recapitulate, as the danger of nephritis is greater during the stage of desquamation, the patient ought to be kept under medical observation for at least three or four weeks. The urine must be examined daily or at least every other day during this period. The routine treatment consists in rest, fluid diet (milk diluted with lime-water, oatmeal water, etc., broths and clear soups). Water in small quantities at regular intervals, with or without salines, should be given. The temperature of the room should be about 72° when possible. Flannels should be worn; in mild cases the above will be sufficient with the addition of fractional doses of calomel four times daily.

If severe headache, nausea and vomiting be present small doses of calomel may be given hourly. Liquid must be given in ʒi doses every five to fifteen minutes until stomach can take more. Coma or convulsions require energetic treatment, as hot-packs, high saline injections, brisk purging, oxygen, caffeine subcutaneously, and the like. Even in coma and when the convulsions have ceased, ʒi doses of water should be given at frequent intervals. The other uremic manifestations yield readily to methods described above. Though the symptoms are frequently alarming, yet if judiciously treated, the tendency is to recovery.

Desperate cases frequently recover. Patients with repeated convulsions or suppression or who by actual measurement may not secrete more than 1 or 2 ounces per day and in whom coma has persisted for twenty-four to thirty-six hours, have been saved despite the unpromising character and apparently fatal prognosis.

A word as to the management of the stage of convalescence. As long as albumin appears in the urine the patient ought to be kept in bed, or, if intractable, at least be confined to the house, in an even temperature, avoiding exposure or a display of too great muscular activity.

The anemia must be combatted by means of light nutrition, food and iron internally. The functions of the bowels may be regulated by the use of salines or calomel.

The myocardial weakness is to be treated by rest and strychnine in appropriate doses.

The diet allowed must be easily digested and light in character.

Plenty of water, a moderate amount of lemonade or carbo-water may be given.

A failure to observe such necessary precautions in this stage is largely responsible for many a chronic nephritis in later years.—*F. Hueber, M.D., New York, in Pediatrics.*

---

### THE COCAINE HABIT.

The cocaine habit is at present attracting considerable attention from a medico-legal standpoint, and while it is a comparatively recent addition to the somewhat lengthy list of "drunk" producers, the easy and insidious manner of its acquirement and development render this form of dissipation difficult of detection. A little investigation into the manner in which this vicious habit is contracted and the pernicious influence it exerts upon its victims and their immediate surroundings will prove edifying to the scientist and surprising to the casual observer

He who is conversant with the classic "Confessions of an English Opium Eater," or has at some time in life experienced the magic spell of the juice of the poppy—yea, even the charming excitation following physiological doses of the dewy distillations of the golden cereals—will more easily comprehend the alluring snares of their more powerful ally, cocaine; more powerful in its immediate influence, more lasting in its remote effects, more potent as a mind-destroyer and crime-breeder than either of the foregoing, it renders its victim a hopeless wreck and leaves but a vestige of that self-respect which once was his in the full fruition of manliness.

A man filled with "tangle-foot" is content with its mollifying influence; however, he is not infrequently seized by an insatiable desire to impart a few drops from his over-filled cup of joy to that division of humanity which never goes beyond buttermilk or seltzer, and thus he sometimes makes the nights of the virtuously inclined hideous by his jocund homeward passage in the "wee small hours." The physiological phantasmagoria of the red glow of the poppy seek the luxurious couch of studied elegance and find solace in the profound sleep of the overfed.

Of the above one may well say, "he who runs may also read,"

but how shall we detect the votaries who worship at the shrine of Erythroxylon? They pass to and fro hourly in the very midst of the busy haunts of life, like a subtle perfume, causing here and there a smile or shrug of the shoulders, but rarely indeed attracting sufficient attention for detection; forsooth, ye merrie "coak" is long on deception and concealment. It is only when the habitué takes more than his usual allowance that he lays himself liable to detection by his extreme affability or disagreeable moroseness, the rapidity with which statements are made and contradicted, the ease with which his most fixed belief concerning ethis, politics or religion may be controverted, together with the purposeless muscular movements and disconnected ideas, all of which, for want of a better name, we will designate as chorea of thought.

The writer has observed no tendency to criminality in the cocaine user so long as he confines himself to this particular form of debauchery. Give whiskey or opium to the case, and we have an irresponsible individual, all the passions unbridled, hope and fear in abeyance, running riotously in our midst without sail or rudder, menacing alike the peace and dignity of the State and the welfare of society.

Since the time when the memory of man runneth not to the contrary, the human species in all climes and conditions has been assiduously engaged in the discovery and consumption of nerve-lulling drugs. The success attendant upon their efforts is fairly familiar to most practitioners of medicine, but the dementia and dwarfing influence on posterity are beyond the comprehension of the human understanding.

The ease with which the cocaine may be obtained is not in keeping with the eternal fitness of things, and scientific bodies are casting about for legislation restricting its sale. The novice timidly approaches the druggist and whispers his desire, but the old-timer advances toward the counter with the eagerness and assurance of a child bent on buying candy, holds up one or two fingers to indicate the amount desired, throws down a nickel or dime, and leaves with the alacrity of a small boy receding from the business end of a wasp, to seek some retired spot and the solace and forgetfulness which the precious purchase will bring.

In enacting statutes for the regulation of the sale of this drug we must move slowly and with that deliberation which will insure the greatest good to the greatest possible number. For instance, it has been suggested that the sales be limited to physicians' prescriptions and be subjected to the statutory provisions regulating the sale of poisons in general.

Now, we will only suppose a case of one of the eighty-five graduates in each one hundred who fail to gain a livelihood in the practice of medicine should turn legally, mind you, to writing prescriptions for these poor, misguided, nerve-wrecked cocaine-thirsting beings. On the other hand we will take the case of a physician owning and operating a drug-store; honestly, do you not think this would be the source of a tempting income and thwart the very object of such legislation by rather increasing than diminishing the sale and consumption of this drug?—*W. D. H., in The Cincinnati Lancet-Clinic.*

---

### OVERWORKED SCHOOL-CHILDREN.

The question of increasing the hours of attendance in the public schools of this city has recently been agitated by those interested in the matter. It is difficult to understand why any one should insist on increasing the number of hours' attendance in our public schools, as they are already sufficient for all practical purposes. The greater part of the work done by the school-children is accomplished at their homes, and longer hours can be of no benefit whatever; on the contrary, must of necessity be a detriment to the children. If there is any one fact thoroughly established in this country it is that we are living too fast. This fast living is enforced upon our children; they are rushed into the school-room at a tender age and compelled to do an excessive amount of work, and no one knows better than the medical man the harmful results of this high-pressure method of education. Time and again it has been our misfortune to witness the wrecking of a young girl as the result of excessive school work. They seem to be less able to stand the strain than boys. Every inducement is held out to those in attendance to reach a maximum, not only in absolute attendance, but in excellency of grading. With many children this is not difficult, while others can only keep up an average by making an extreme effort, and this effort, day after day, finally results in wrecking their physical constitution. If there was any necessity, or even apparent necessity, for all of this rush and high-pressure work there might be some excuse for it, but there is none, and the people who are advocating it should know better. They have been observers for years, and have seen the results of high-pressure work, not only in females but among males, and as the school is supported by taxpayers they should have the right to say what they want concerning the physical management of their children. We are of the opinion that no good whatever will result from increasing the length of the study hours in our public schools. As a teacher of medicine for more than a quarter of a century we have been a very close observer of the results of high-pressure work among students, and feel confident that in our medical schools the pressure is too great for the average physical man—too many working hours and too much outside work to keep up with the recitations—and yet the working hours in the medical schools are, comparatively speaking, fewer than those of the public schools. More play and less work on the part of the average child in attendance at the public schools will result in the development of a better class of men and women than are being developed to-day.—*American Practitioner and News.*

---

### THE UNRECOGNIZED CHANCRE. GOTTHEIL.

(Author's Abstract.)

The Unrecognized Chancre. In the *International Medical Magazine* for October William S. Gottheil calls attention to the frequent insignificance and fugacity of the syphilitic initial lesion, which leads to its non-recognition in quite a large proportion of cases. Ignorance of its occurrence, and not voluntary falsification, is the cause of the frequent absence of a syphilitic history in undoubtedly specific cases. The author calls attention to the following points of diagnosis:

1. The presence of a tumor as the original lesion. In its essence, and invariably at the beginning, the chancre is a small round cell accumulation in the skin or subcutaneous tissue. Ulceration may occur, and usually does, or even phagadenism; but these are accidental, and epiphenomena, and almost invariably the specific induration is appreciable at the base of the lesion.

2. The tumor is indolent, painful, and recalcitrant to treatment.

3. A peculiar and characteristic "stony" induration of the nearest lymphatic glands accompanies it, different from the general adenopathy that occurs later as a consequence of the systemic infection. Other lesions, as gummata, do not show it.

4. Chancre runs its full course in a few weeks, whilst tuberculosis takes months, and carcinoma even years, for its development.

5. The well known signs of general luetic infection, osteocopic pain, cephalalgia, synovitis, general lymphadenitis, exanthem, etc., must be carefully and persistently searched for in every suspicious case. They may be so slight as to entirely escape careless examination.

---

### METHYLENE-BLUE IN URETHRITIS.

It is best given in gelatine capsules in one-grain doses three or four times a day. After the fourth day the dose may be reduced to twice a day. Given alone it sometimes causes irritation of the neck of the bladder, but when combined with the oil of nutmeg there is no trouble of this kind. Oil of sandalwood is a desirable adjuvant because of its diuretic action and also on account of its sedative effect upon inflamed mucous membrane. Recent observations show that, when given internally, methylene-blue reappears unchanged in the urine within two hours. By giving four one-grain doses of methylene-blue daily there is always enough of it in the urine to kill all the germs it comes in contact with. This is irrigation "from above," irrigation, not of the urethra alone, but of the entire urinary tract. By this method of irrigation there is no danger of forcing the infection into remote recesses of the genito-urinary organs.

Troublesome gastric symptoms sometimes follow the administration of the methylene-blue of the shops, but, with the following formula put up in elastic capsules, uniformly satisfactory results have been personally obtained:

| | | |
|---|---|---|
| ℞ | Methylene-blue | 1 grain. |
| | Oil of nutmeg | 1 drop. |
| | Oil of sandalwood | 2 drops. |

The above formula should not be used for more than ten days without intermission, and while giving it the patient should be instructed to drink freely of water.—*The Medical Times.*

### ACETANILID POISONING.

Cases of poisoning with acetanilid are not very infrequent, especially in children. Dr. Philip Brown (*Am. Jour. Med. Science*, cxxii, 7) reports a fatal case. The patient, a man of 37, took six "headache powers," each containing 10 grn. of acetanilid. He was found delirious, complained of abdominal pain, vomited and was slightly jaundiced. He was taken to a hospital; his temperature rose to 100.2° F., the lips and nails became intensely cyanotic, respirations shallow and frequent. The urine, of which 10 oz. were passed on admission, was nearly black in color and strongly alkaline in reaction. After the second day complete suppression of urine supervened, and six days later the man died.

Contrary to current conceptions, the patient showed general hyperesthesia, not anesthesia, the reflexes were increased and sensory as well as motor functions retained to the end. Acute nephritis, acute progressive jaundice and hemorrhage from the bowels were also present in this case.—*Merck's Archives*, February, 1902.

### THE FEE-SPLITTERS.

The abominable practice of fee-splitting must come to an end. The fellow who auctioneers his patients off to the surgeon and specialist—to the highest bidder—has had his day, and will as surely receive his just deserts as time lasts. At the next meeting of the American Medical Association there can be no doubt but what that great body will take such action as will force every one of the fee-splitters to abandon the abominable practice or be expelled from any medical society that they may then belong to, and will bar them from entering any medical societies in the future. There can be no way of evading the punishment that is in sight for them; it is inevitable.—*American Practitioner and News.*

